# Investigating the Mechanism of Inhibition of Cyclin-Dependent Kinase 6 Inhibitory Potential by Selonsertib: Newer Insights Into Drug Repurposing

**DOI:** 10.3389/fonc.2022.865454

**Published:** 2022-05-26

**Authors:** Mohammad Hassan Baig, Mohd. Yousuf, Mohd. Imran Khan, Imran Khan, Irfan Ahmad, Mohammad Y. Alshahrani, Md. Imtaiyaz Hassan, Jae-June Dong

**Affiliations:** ^1^ Department of Family Medicine, Gangnam Severance Hospital, Yonsei University College of Medicine, Seoul, South Korea; ^2^ Centre for Interdisciplinary Research in Basic Sciences, Jamia Millia Islamia, New Delhi, India; ^3^ Department of Internal Medicine, Gangnam Severance Hospital, Yonsei University College of Medicine, Seoul, South Korea; ^4^ Department of Molecular Biology, Beykoz Institute of Life Sciences and Biotechnology, BezmialemVakif University, Istanbul, Turkey; ^5^ Department of Clinical Laboratory Sciences, College of Applied Medical Sciences, King Khalid University, Abha, Saudi Arabia

**Keywords:** drug repurposing, cyclin-dependent kinases, anticancer therapy, MD simulation, molecular docking, drug design and development

## Abstract

Cyclin-dependent kinases (CDKs) play significant roles in numerous physiological, and are considered an attractive drug target for cancer, neurodegenerative, and inflammatory diseases. In the present study, we have aimed to investigate the binding affinity and inhibitory potential of selonsertib toward CDK6. Using the drug repurposing approach, we performed molecular docking of selonsertib with CDK6 and observed a significant binding affinity. To ascertain, we further performed essential dynamics analysis and free energy calculation, which suggested the formation of a stable selonsertib-CDK6 complex. The *in-silico* findings were further experimentally validated. The recombinant CDK6 was expressed, purified, and treated with selonsertib. The binding affinity of selonsertib to CDK6 was estimated by fluorescence binding studies and enzyme inhibition assay. The results indicated an appreciable binding of selonsertib against CDK6, which subsequently inhibits its activity with a commendable IC_50_ value (9.8 μM). We concluded that targeting CDK6 by selonsertib can be an efficient therapeutic approach to cancer and other CDK6-related diseases. These observations provide a promising opportunity to utilize selonsertib to address CDK6-related human pathologies.

## Introduction

Cyclin-dependent kinases (CDKs) are a large family of heteromeric serine/threonine protein kinases that play a crucial role in cell cycle progression (1). CDKs are involved in different biological processes, including transcription, translation, neurogenesis, and apoptosis (2). Dysregulation of CDKs is directly associated with oncogenesis (3, 4). The transient activation of CDKs by forming a complex with different cyclin proteins regulates cell cycle progression (5). About 20 CDKs and 30 cyclins have been reported so far. CDK1, CDK2, CDK4, and CDK6 are involved in the transition of cell cycle phases, whereas CDKs 7-11 regulate the transcription (6).

The CDK6 gene is located on Chromosome 7 and translates into a 326 amino-acids protein (7). The CDK6-cyclin D complex phosphorylates retinoblastoma (Rb) protein leading to E2F transcription factors activation (8). Activated E2Fs trigger the regulatory genes, including cyclin E, which ensure the irreversible transition of G1 to S phase in cell-cycle progression (9). In addition, CDK6 plays a transcriptional role in tumor angiogenesis and phosphorylates nuclear factor kappa-B (NF-κB), thereby linking cancers to inflammation (7, 10). Different components of the CDK6-cyclin D complex are altered in various malignancies and neurodegenerative disorders (11, 12).

Studies have reported an increased expression of CDK6 in leukemia, T-cell lymphoblastic lymphoma, and B-lymphoid malignancies (13–15). Increased activity of CDK6 is responsible for the metabolic switching in energy consumption pathways, leads to activation of alternative pathways that inhibit the production of reactive oxygen species (ROS), and prevents apoptosis in cancer cells (16–19). The overexpression of CDK6 initiates the multidrug-resistant gene that favors the growth and development of cancer cells and protects the cells from apoptosis (20, 21). The studies confirm the crucial role of CDK6 in cell cycle regulation and metabolism. Furthermore, the overexpression of CDK6 is also widely investigated to be associated with diabetes and inflammatory diseases (22). All the research findings favor the targeting of CDK6 for the successful management of various diseases, which led to the discovery of reliable CDK6-targeted drugs (23, 24). Palbociclib, ribociclib, and abemaciclib are highly selective and reversible inhibitors of CDK6 that interact with the ATP binding pocket of CDK6 and form hydrogen bonds and are used for the treatment of cancer (25). In enzyme assays, all three compounds show different potency in the activity against CDK6. Palbociclib, Ribociclib, and abemaciclib showing IC50 values 16 nM, 39 nM and 10 nM, respectively.

Selonsertib is a recently developed potential and selective inhibitor of apoptosis signal-regulating kinase-1 (ASK-1) with efficient anti-inflammatory, anti-fibrotic, and anti-neoplastic activities (26–28). It plays a key role in hepatocyte injury, inflammation, cellular proliferation, and fibrosis in non-alcoholic steatohepatitis (NASH). However, recent studies have shown that selonsertib failed to show an anti-fibrotic effect in NASH during clinical trials (26, 29). Selonsertib is a serine/threonine-protein kinase inhibitor that reverses the multidrug resistance properties of cancer cells by inhibiting the overexpression of ATP-binding cassette (ABC) transporters and reducing the proliferation of cancer cells (30). When orally administered, selonsertib binds competitively (ATP-competent) to the catalytic domain of ASK1, thus averting its phosphorylation and activation (28, 31). Further, the binding inhibits the phosphorylation of downstream kinases, viz. p38 mitogen-activated protein kinase (p38 MAPK) and c-Jun N-terminal kinases (JNKs) (28). CDK6 has involved cancer progression *via* (RB)-E2F signaling. An uncontrolled regulation of the cyclin D-CDK4/6-INK4-RB pathway has been reported in cancer which causes uncontrolled cell cycle and cell growth. Although selonsertib had dose-dependent effects indicating good pharmacodynamic activity, we can employ its excellent drug-like features to treat other diseases. However, selonsertib has not been investigated for its inhibitory potential against CDK6 (32). 

Since CDK6 is considered an attractive drug target for cancer therapy, we aimed to see the CDK6 inhibitory potential of Selonsertib (33). We hypothesized that selonsertib binding to CDK6 may be a reasonable therapeutic approach toward cancer management. Our group has been working toward developing new therapeutics and exploring the possibility of repurposing existing molecules as a CDK6 inhibitors (34). Here, we used computational and experimental methods to investigate the binding affinity and enzyme inhibitory potential of selonsertib against CDK6. Thus, we report Selonsertib as a CDK6 inhibitor for the first time, which may be implicated in cancer control and prevention. Our findings have great potential in designing and developing a new class of potent CDK6 inhibitors from an already available pool.

## Materials and Methods

### Molecular Docking

Molecular docking studies were carried out to better understand the binding mode and the binding affinity of selonsertib against CDK6. The crystal structure of human cyclin-dependent kinase 6 complexes with a flavonol inhibitor, fisetin, was downloaded from the RCSB protein data bank (1XO2) (35). On the other hand, 3D structure of selonsertib was retrieved from the PubChem compound database (PubChem id: 71245288). The molecular docking was performed using InstaDock (36). Before conducting the molecular docking experiment, all the HETATM and water molecules already present within the structure of CDK6 were removed. A total of 20 runs were performed using the Lamarckian genetic algorithm. Of the 20 conformations generated, the best one was selected based on binding free energy. The visualization of the complex was done using PyMOL.

### Molecular Dynamics Simulations

To better understand the binding of selonsertib within the active site of CDK6, we performed a 100 ns molecular dynamics (MD) study for this complex (37, 38). The docked complex of CDK6-selonsertib prepared using molecular docking was taken as a starting point for MD study as described (39, 40). We used the GROMACS 4.6.7 package with the gromos96 force field to perform the MD simulation (41). GROMACS is a widely used tool for performing MD simulation studies, and its utilization in protein-ligand simulation has been reported in many studies (42, 43). The CDK6-selonsertib complex was solvated within the dodecahedron box of an explicit SPC water model with 0.1 nm margin between the box walls and solute. Na+ or Cl− counterions were added to neutralize the system charge. The particle-mesh Ewald method (cutoff distance of 0.1 nm) was employed to calculate long-range electrostatic interactions. Lennard-Jones 6–12 potential was used for evaluating the van der Waals interactions. For this calculation, the cutoff distance was set to 0.1 nm. The LINCS algorithm constrains bond lengths while setting the time step to 0.002 ps.

Further energy minimization was performed using the steepest-descent method for 10,000 steps to remove the steric clashes between atoms. The whole system was further subjected to equilibration for 1 ns. To maintain the system at 300 K and 1 atm, Berendsen weak coupling systems were utilized. Maxwell Boltzmann distribution was used for randomly generating the Initial velocities. The final 100ns production run was performed at 300 K in NPT ensemble. Furthermore, xmgrace was used to generate graphs (http://plasmagate.weizmann.ac.il); PyMol and VMD were utilized for further graphical inspections and analysis.

### Principal Component Analysis

Principal component analysis (PCA) was performed and analyzed to investigate the collective motions in protein (44). The covariance matrix, C, was calculated using the following equation:


$$C_{ij}=<(x_{i}-<x_{i}>)(x_{j}-<x_{j}>)$$

Where xi and xj are the instant coordinates of the ith and jth atoms of the system, while <xi> and < xj> represent an ensemble average.

### Free Energy Landscape

The Free energy landscape (FEL) was analyzed to understand the stability of docked complex (45). The FEL was depicted as:


$$\Delta G(X)=K_{B}T\;\text{In}\;P(X)$$

Where Boltzmann constant is denoted by KB, T is the absolute temperature, while the probability distribution of the molecular system along the Pc is denoted by P(X).

### Binding Free Energy Calculation

Molecular mechanics (MM)-Poisson–Boltzmann surface area (PBSA) (MM-PBSA) approach plays a more efficient role in drug discovery than the traditional free energy calculations (46, 47). The binding free energy was calculated by considering the vacuum potential energy and solvation free energy (polar and nonpolar). The polar and nonpolar solvation energy terms were estimated using the Poisson–Boltzmann equation and solvent accessible surface area (SASA) methods. The Poisson–Boltzmann equation approximates the electrostatic component of biological macromolecules and helps study the ligand-binding affinity of the protein. The SASA method helps identify the protein’s surface with van der Waals contact probed by the solvent sphere. The MMPBSA.py module was used to perform the MM-PBSA calculations using the AMBER software.

This approach calculates the binding free energy (Δ*G*_binding_) according to the following equations:


(1) $$\Delta G_{binding}=\Delta G_{MM(Potential\;energy\;in\;vaccum)}\;+\Delta G_{sol\;(solation\;effects)}$$

where


(2) $$\Delta G_{MM}=\Delta G_{coulomb\;(electrostatic\;interaction)}\;+\Delta G_{Vdw}$$

and


$$\Delta G_{sol}=\Delta G_{polar}+\Delta G_{nonpolar}$$

### Expression and Purification of Recombinant CDK6

The CDK6 gene was cloned successfully within the pET28a+ vector, confirmed by the gene-sequencing method. We have cloned the CDK6 gene in the pET28a+ vector and subsequently transformed the Codon+ competent cells to express CDK6 protein induced by IPTG. The overexpressed protein was purified using our optimized protocol using Ni-NTA column chromatography (48, 49). Purified protein was confirmed by 12% SDS-PAGE and Western blot as described (50).

### Measurement of Binding Affinity of Selonsertib With CDK6

Fluorescence measurements were performed on Jasco spectrofluorimeter (Jasco, Tokyo, Japan Model FP-8250) at 25 ± 0.1°C maintained by an external thermostat Peltier device. Selonsertib was initially dissolved in DMSO and then diluted 100 times to make a working solution of 50 μM concentration 50 mM Tris buffer containing 150 mM NaCl. We made a protein solution of 4 μM and titrated it with successive addition of selonsertib in 1 cm quartz cuvette. The protein solution was excited at 280 nm, and the fluorescence emission spectrum was recorded in the range of 300-400 nm. After deducing the corresponding concentration of selonsertib as blank, the resultant fluorescence emission spectra were taken for the subsequent calculation. We have plotted fluorescence intensity at λ_max_ [Selonsertib, μM] and fitted it to the modified Stern-Volmer equation to obtain binding constant (*K*_a_) and the number of binding sites (*n*) per molecule as described in our previous communications (51, 52).

### Enzyme Inhibition Assay

ATPase assay measured the free form of phosphate release after the hydrolysis of ATP as described (34, 53). At the same time, the kinase assay measured the protein kinase activity. A protein kinase can transfer an inorganic phosphate from ATP to another specific molecule. This study demonstrates the effect of selonsertib on CDK6 kinase activity and found that selonsertib significantly inhibits the CDK6 kinase activity. The enzyme activity of CDK6 was confirmed by a Malachite green-based microtitre-plate assay (BIOMOL^®^ Green reagent, Enzo Life sciences). About 2 μM of CDK6 protein was incubated with assay buffer (20 mM Tris-HCl and 100 mM NaCl; pH 8.0 with 10 mM MgCl_2_ and increasing concentrations of ATP at 25°C for 30 minutes. After adding the Malachite green reagent to the reaction mixture, the system was incubated for 20 minutes until the appearance of color was measured at 620 nm on a multiplate ELISA reader. The free inorganic released from ATP was estimated for kinase activity using the standard phosphate curve. After confirming the CDK6 activity, a similar experiment set of 2 μM protein was incubated for 60 minutes with an increasing concentration of selonsertib in a 96-well plate at 25°C. Subsequently, 10 mM MgCl_2_ and 150 μM freshly prepared ATP was added to the protein solution. After 30 minutes, BIOMOL^®^ reagent was added to the reaction to terminate the enzyme reaction, and absorbance was measured at 620 nm after 20 minutes. All reactions were performed in triplicates. The inhibitory enzyme potential of selonsertib was calculated in terms of % inhibition using our previously described protocol (34, 49). In brief, the raw data were converted to % inhibition values using the formula 100 – (A/A0 × 100) where A0 and A represent enzyme activity of CDK6 in the absence and presence of Selonsertib. The percent inhibition in kinase activity was plotted against log [compound], and data were fitted to estimate the value of IC50 (50% of ATPase inhibition) for Selonsertib using GraphPad Prism 5.0.

## Results

### Molecular Docking

**Figure 1:**
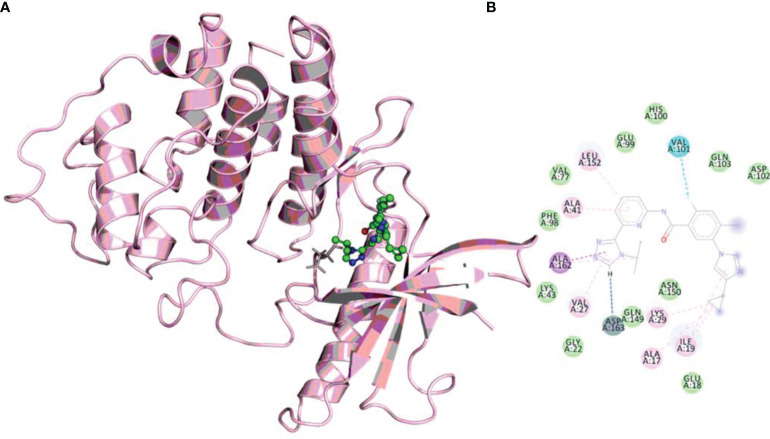
**(A)** The docked complex of selonsertib within the active site of CDK6. **(B)** Showing interactions of active site residues of CDK6 with the selonsertib.

The molecular docking method helps predict a compound’s binding orientation within the receptor’s binding pocket and its consequent binding affinity (54–56). Docking of selonsertib with the CDK6 shows a promising score and excellent binding affinity. To get atomistic insights into the binding pattern of selonsertib with the CDK6, we performed a structural analysis of docked complex. The analysis of the docked complex of CDK6-selonsertib shows that selonsertib is tightly bound within the active site cavity of CDK6. The estimated binding affinity of selonsertib was −10.9 kcal/mol (**Table 1**). In the CDK6-selonsertib complex, we observed the active site residues of CDK6, Ala17, Ile19, Val27, Ala41, Lys43, Val77, Val101, Gln103, Ala162, Lue152, and Asp163 are prominently involved in selonsertib binding (**Figure 1**). These residues are mainly involved in the ATP binding and kinase activity of CDK6. Interestingly, the CDK6-selonsertib complex was stabilized by several hydrophobic interactions, while Asp163 was the active residue of CDK6, making a hydrogen bond with the selonsertib. Thus, the formation of a strong complex of selonsertib with the CDK6 interferes with the substrate accessibility and thus predominantly inhibits its kinase activity.

**Table 1:** List of interactions of selonsertib against CDK6.

Interactions	Distance	Interaction Category	Interaction Type
LIG301:H21 - ASP163:OD2	2.61641	Hydrogen Bond	Carbon Hydrogen Bond
VAL101:O - LIG301:F1	2.44786	Halogen	Halogen (Fluorine)
GLN103:O - LIG301:F1	3.60412	Halogen	Halogen (Fluorine)
ALA17 - LIG301	4.92137	Hydrophobic	Alkyl
LIG301 - ILE19	4.98136	Hydrophobic	Alkyl
LIG301:C14 - VAL27	4.63574	Hydrophobic	Alkyl
LIG301 - ILE19	5.29688	Hydrophobic	Pi-Alkyl
LIG301 - VAL27	3.80786	Hydrophobic	Pi-Alkyl
LIG301 - ALA162	4.06387	Hydrophobic	Pi-Alkyl
LIG301 - ALA41	3.72959	Hydrophobic	Pi-Alkyl
LIG301 - VAL77	5.47874	Hydrophobic	Pi-Alkyl
LIG301 - LEU152	5.27223	Hydrophobic	Pi-Alkyl
LIG301 - ALA162	4.75756	Hydrophobic	Pi-Alkyl

### Molecular Dynamics Simulation

**Figure 2:**
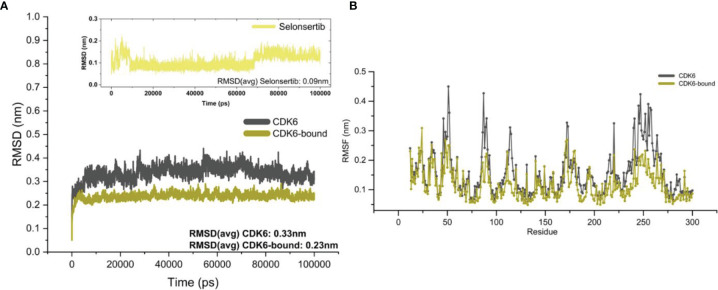
Structural fluctuations in the CDK6 was evaluated by MD simulation. **(A)** Backbone RMSD values of the unbound CDK6 protein and CDK6 protein complexed with selonsertib. The RMSD of selonsertib is shown in the inset **(B)** RMSF value of the unbound CDK6 protein and CDK6 protein complexed with selonsertib.

To gain insights into the structural fluctuations of CDK6 upon binding selonsertib, we performed MD simulation studies. The structural features that were evaluated during MD analysis are root mean square deviation (RMSD), root mean square fluctuation (RMSF), and radius of gyration (*R*g) as described (40, 57, 58). MD simulations of CDK6 in the free and complex with selonsertib were performed for 100 ns. **Figure 2A** shows that the CDK6-selonsertib complex form is almost stable throughout the 100 ns trajectory. After the binding of selonsertib, the RMSD of backbone atoms of CDK6 was more stabilized. When evaluating the distributions between ligand and protein, initial 10ns of trajectory are sufficient for the complex’s equilibration. A little fluctuation was recorded, but these minute fluctuations in small globular proteins are negligible. The RMSD was less than 0.3 nm for the complex for the total trajectory analysis. The average RMSD for the unbound and complexed was also evaluated. The average RMSD was 0.33 nm for the unbound form, which was reduced to 0.23 nm for the selonserib bound complex. The complex’s RMSD suggests the complex’s stability during the entire simulation period (59). Moreover, the fluctuations in the RMSF values were found during simulation in the structure containing CDK6 bound to selonsertib **(** **Figure 2B** **)**.

**Figure 3:**
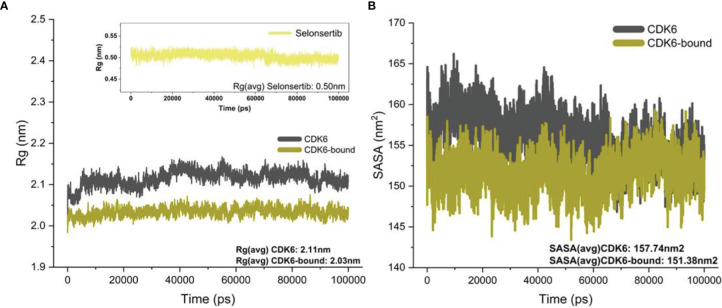
**(A)** *R*g of the backbone carbon alpha for the unbound CDK6 protein and CDK6 protein complexed with selonsertib. **(B)** The SASA (nm^2^) for the unbound CDK6 protein and CDK6 protein complexed with selonsertib during the 100 ns.

The *R*g value defines the atom distribution around a given protein axis, which is an important parameter to determine the backbone atom’s stability and integrity (60–62). We calculated *R*g values of CDK6 in the presence and absence of selonsertib. **Figure 3A** shows the fluctuation in the *R*g of CDK6 in the free and selonsertib bound form, indicating a stable complexation throughout the simulation trajectory. A close analysis shows that the selonsertib bound form of CDK6 was shown comparatively less fluctuation in the *Rg*. The average *R*g values of both structures were evaluated. It was found that the selonsertib bound structure of CDK6 has an average *R*g value of 2.11 nm^2^, which was comparatively less than its free form (2.03 nm^2^). This clearly shows CDK6 becomes more compact after the binding of selonsertib. The findings of this study indicate the stable binding of the CDK6-selonsertib complex with negligible atomic fluctuations, exhibiting the complex to be stable.

Furthermore, the solvent-accessible surface area (SASA) was studied to evaluate the protein’s structural folding–unfolding dynamics under the solvent environment by studying its hydrophobic core and solvent accessibility (63). We plotted the SASA to investigate the effect of selonsertib on the solvent accessibility of CDK6 (**Figure 3B**). The SASA plot shows that the binding of selonsertib affects the SASA value significantly compared to unbound systems. The average SASA value for CDK6 was 157.74 nm^2^, while for the CDK6-selonsertib complex, it was 151.38 nm^2^. The decrease in the SASA after the binding of selonsertib signifies the stabilized protein structure after the binding of selonsertib **(** **Figure 3B** **).** Overall findings of the MD simulation studies indicate that CDK6 forms a stable complex with selonsertib.

**Figure 4:**
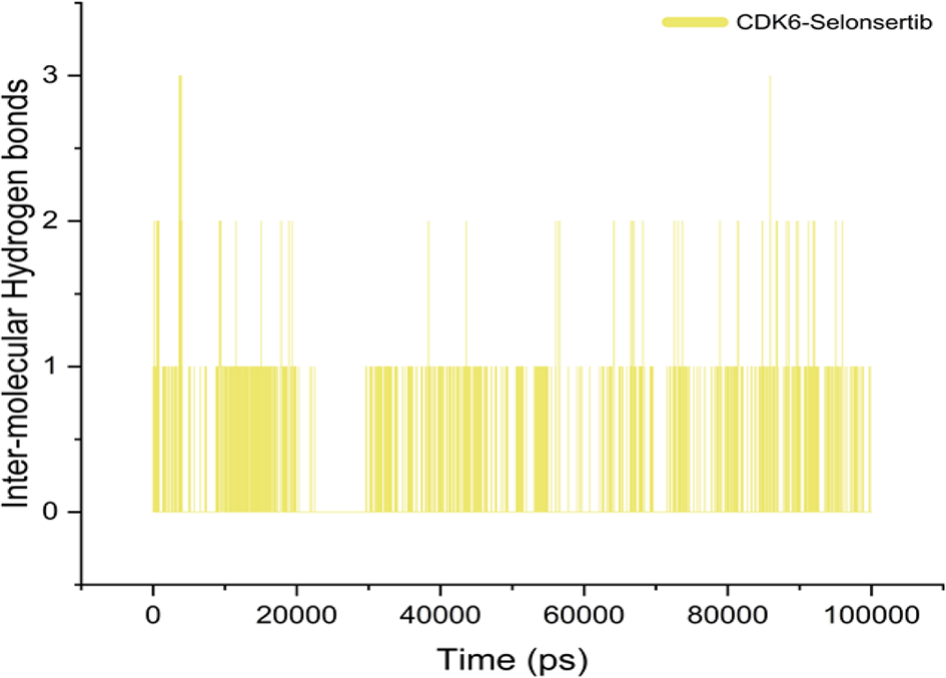
Intermolecular H-bonds in the Selonsertib-CDK6 complex during MD simulation.

Although various interactions facilitate ligand binding to its target protein, hydrogen bond formation is a crucial role player in complex stabilization (40, 64, 65). The higher number of hydrogen bonds in the ligand-protein complex is responsible for the stability of complex and strong ligand affinity. Selonsertib forms an average of one bond throughout the simulation period. This indicates that the binding was governed mainly by hydrophobic interactions **(** **Figure 4** **)**.

### Principal Component Analysis

**Figure 5:**
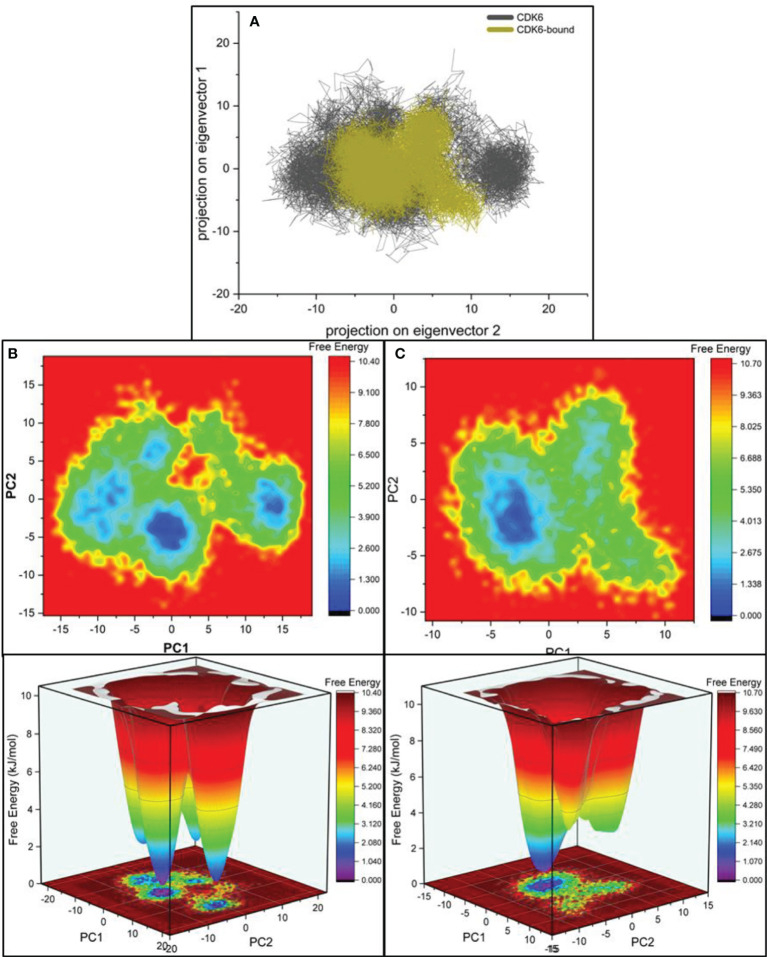
**(A)** Projections of the eigenvectors for PCA analysis of CDK6 complexed with selonsertib. Free energy contour for the **(B)** CDK6 and **(C)** selonsertib bound structure of CDK6.

We performed principal component analysis (PCA) to evaluate and discriminate the conformational changes resulting from pressurization and thermal fluctuations (66–68). The biggest eigenvectors from PCA depict the rigorous atomic motion in the protein (69–71). We investigated the projection of eight eigenvectors for the PCA of CDK6 bound with selonsertib **(** **Figure 5A** **)**. The trajectory suggested largely similar atomic motions during the simulation. The PCA plot of eigenvalues along the eigenvectors was projected. The results show that the selonsertib bound structure of CDK6 occupies a smaller conformational space, indicating higher structural stability than its apo form (**Figure 5A**). Further, to understand the protein-folding pattern differences between the apo and selonsertib bound form of CDK6, we plotted the free energy landscapes **(** **Figures 5B, C** **)** and found that most of the simulation ensembles in the selonsertib bound structure are concentrated to a narrow range of conformational space. These observations suggest a better stability and compact packing of the selonsertib bound structure.

### Molecular Mechanics Poisson–Boltzmann Surface Area

We further performed Molecular mechanics Poisson–Boltzmann surface area (MM-PBSA) analysis for the selonsertib bound CDK6 complex (57). The MD trajectories were used to calculate various thermodynamics parameters involved in the complex formation. The most important parameter was calculating the binding free energy of the complex during the simulation period (**Table 2**). The binding free energy of selonsertib against CDK6 was −18.09 (± 0.36) kcal/mol, indicating a strong binding affinity.

**Table 2:** Binding free energy and interacting amino acid residues in the docked CDK6 and selonsertib complex.

Molecule	Δ*G*	VDWAALS	EEL	Δ*G*gas	Δ*G*solv
	Avg (Std. Err. of Mean) (kcal/mol)	Avg (Std. Err. of Mean) (kcal/mol)	Avg (Std. Err. of Mean) (kcal/mol)	Avg (Std. Err. of Mean) (kcal/mol)	Avg (Std. Err. of Mean) (kcal/mol)
**CDK6-selonsertib**	-18.09 (± 0.36)	-44.87 (± 0.33)	-26.29 (± 0.69)	-71.16 (± 0.72)	53.07 (± 0.66)

### Expression and Purification of Recombinant CDK6

**Figure 6:**
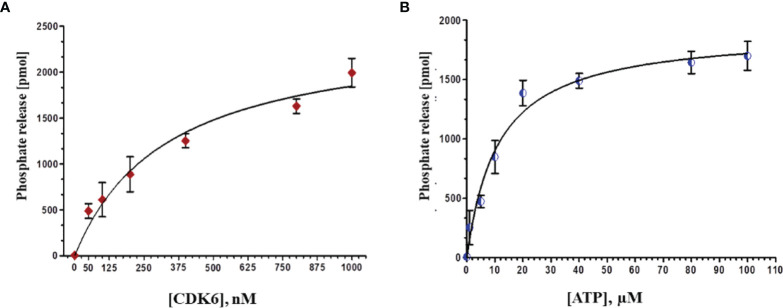
**(A)** Phosphate release (pMol) with increasing concentrations of protein (0-1000 nm) at fixed ATP concentration (20 µM). **(B)** With increasing concentrations of ATP (0-100 µM) at constant protein concentration (500 nM).

The recombinant CDK6 protein expressed in *E. coli* (codon^+^) cells induced by the IPTG. The overexpressed protein in the form of inclusion body was solubilized by N-Laurosyl sarcosine. After centrifugation, the supernatant was subjected to Ni-NTA affinity chromatography and bound protein was eluted with the help of increasing concentrations of imidazole. CDK6 protein was eluted at 500mM imidazole concentration. The purity of CDK6 was confirmed by SDS-PAGE, which showed a single protein band at ∼37 kDa (data not shown). Further, the enzymatic activity of recombinant CDK6 protein was performed by ATPase assay suggesting the excellent activity in refolded purified protein **(** **Figure 6** **)**.

### Fluorescence Measurements

**Figure 7:**
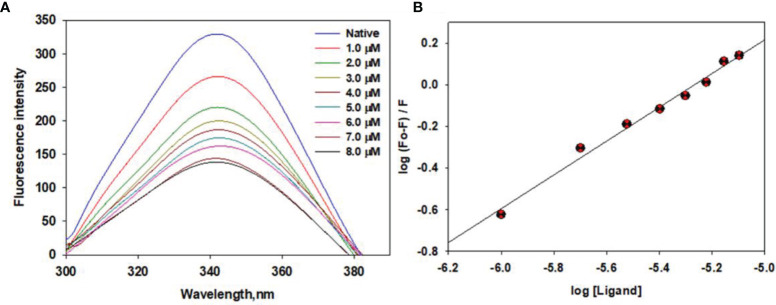
Fluorescence binding studies of selonsertib with CDK6. **(A)** Fluorescence emission spectra of CDK6 with increasing concentrations of selonsertib (1–8 μM). **(B)** Modified Stern–Volmer plots to estimate the binding affinity of selonsertib with the CDK6.

We estimated the binding affinity of selonsertib with CDK6 using standard fluorescence measurements. The CDK6 concentration was optimized at 4 µM and titrated with increasing Selonsertib concentrations from a solution of 1.0 mM stock. The fluorescence emission spectra were recorded at 300-400 nm by keeping the excitation wavelength fixed at 280 nm. The final concentration of selonsertib was varied from 1 to 8 μM to achieve the saturation point. **Figure 7** shows a significant decrease in fluorescence emission spectra of CDK6 with increasing concentrations of selonsertib **(** **Figure 7A** **)**. A notable decline in fluorescence intensity with each titration step indicates a significant binding affinity of selonsertib with the CDK6. The fluorescence quenching data were fitted to the modified Stern-Volmer equation to obtain the binding constant (*K*_a_) and the number of binding sites per CDK6 molecule (*n*) **(** **Figure 7B** **)**. The obtained binding constant values were 1.8x10^5^ M^-1,^ and the number of binding sites per CDK6 molecule (*n*) was 1.

### Enzyme Inhibition Assay

**Figure 8:**
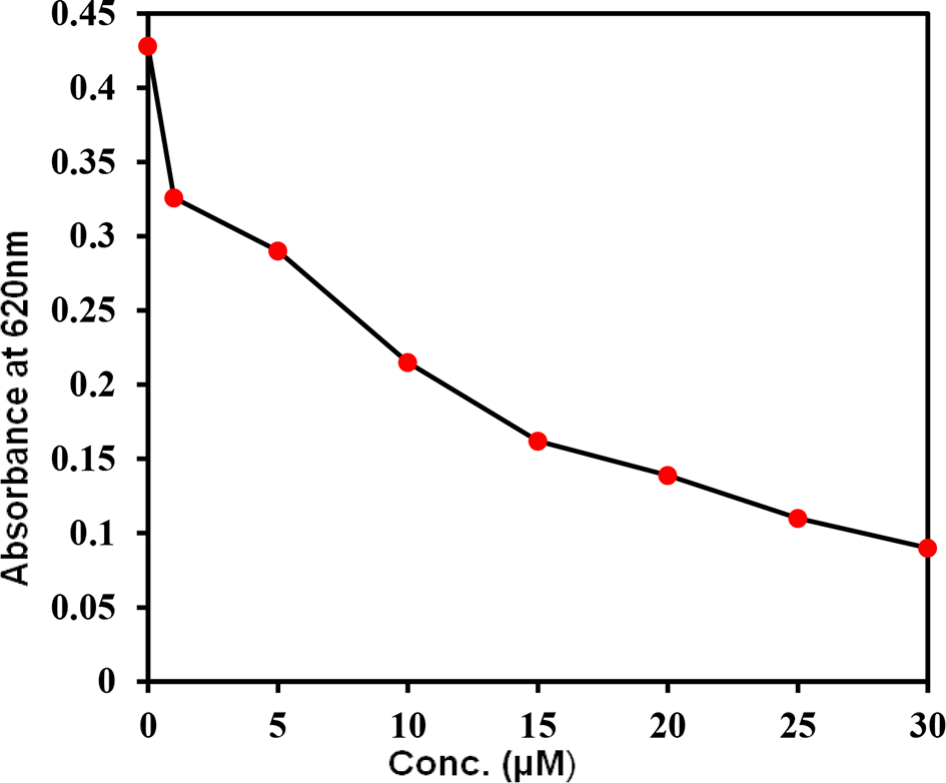
Kinase activity of CDK6 with increasing concentrations of selonsertib. The kinase activity of CDK6 was measured in the form of ATP release (ATPase activity).

The kinase activity of CDK6 was measured with increasing concentrations of selonsertib to calculate the IC_50_ value. **Figure 8** shows the amount of inorganic phosphate released by CDK6 with increasing \selonsertib concentration. We observed that the binding of selonsertib to CDK6 inhibits its kinase activity **(** **Figure 8** **).** The data of enzyme inhibitory potential of selonsertib with increasing concentration were plotted to calculate the IC_50_ value. We estimated the IC_50_ value of selonsertib with CDK6 as ~9.8 μM using AATBioquest software. These findings clearly indicate that the strong binding affinity of selonsertib to the CDK6 causes a significant decrease in its enzyme activity. Thus, selonsertib could be implicated as a potential CDK6 inhibitor.

## Discussion

The activation of signaling cascades is an anomalous recurrent event occurring in a range of human cancers (72, 73). Since most protein kinases are fundamental components of nearly all signaling pathways, the development of anticancer therapies targeting these vital enzymes has always gained interest among researchers (74–76). The majority of protein kinases, when amplified, over-expressed, or constitutively active, stimulate the proliferation, growth, survival, and migration of cells, thereby assuming the oncogenic properties. CDKs, along with their analogous cyclin, orchestrate the complex events regulating the cell cycle (2, 77). Usually, in cancer cells, the activity of CDK-cyclin complexes is deregulated, thereby resulting in uncontrolled cell growth owing to increased Rb phosphorylation (Rb inactivation) and transcriptional activity (78, 79). Thus, targeted kinase inhibition is a reasonable therapeutic approach (80–82).

In the last few decades, various efforts have been devoted to developing small molecules that selectively/specifically inhibit protein kinases (83, 84). Rapidly emerging data with selective inhibitors of cell cycle kinases have corroborated them as anticancer drug targets, upholding enduring preclinical prediction. Selonsertib, a selective inhibitor of ASK-1, possesses efficient anti-inflammatory, anti-fibrotic, and anti-neoplastic activities (28). It plays a vital role in preventing inflammatory cytokine production, down-regulation of the fibrotic gene expression, inhibition of cellular proliferation, and suppression of excessive apoptosis (85).

Our molecular dynamics simulation study focused on the dynamic state of CDK6 in the apo form and the effect of selonsertib binding **(** **Figures 2**, **3** **)**. The RMSD values, which measure the structural stability, came out to be 3Å for the CDK6 protein. We further analyzed the performance of the ligand at the binding pocket and found it to be highly stable in the complex. Selonsertib, the proposed lead, when bound to CDK6, showed an RMSD value below 1.5Å. Residual RMSF analysis for the unbound and bound form follows the same trend as RMSD. The residues fluctuation for the protein in the complex form showed reduced movement consistently and homogeneously. We measured the difference in compactness by measuring the radius of gyration throughout dynamics. The CDK6 protein complex with Selonsertib has a radius of gyration values lower than the protein alone.

The essential dynamic plot of eigenvectors for the bound and unbound states drew parallelism with our previous biophysical results. As evident from **Figure 5**, the CDK6 in the bound state has reduced dynamic behavior, having ensembles concerted defined by well-defined minima. To understand the packing behavior of the protein in both states, we measured the intramolecular hydrogen bond. The difference was not significant, suggesting no major secondary structural shift. The intramolecular hydrogen bonds between the CDK6 residues and the selonsertib were found at an average of one formed by the residues D163. Most of the binding interactions were nonionic, involving residues A17, I19, V27, A41, V77, V101, N103, L152, and A162. These biophysical results suggest Selonsertib is an effective inhibitor against the inflammatory cytokine of interest, CDK6.

Here, we evaluated the inhibitory potential of selonsertib on CDK6. It was observed from the fluorescence binding studies that the binding affinity of selonsertib toward CDK6 was efficient. Selonsertib is reported as a potent and highly selective ATP-competitive inhibitor ASK1 with a pIC50 value of 8.3 (86). The role of selonsertib as a CDK6 inhibitor was further evaluated by ATPase activity. ATPase activity of CDK6 in the presence of Selonsertib shows an IC_50_ of 9.8 μM. Previously, we reported some natural products as CDK6 inhibitors and found the IC50 value of Selonsertib with CDK6 is quite comparable with these natural products, such as vanillin, quercetin, and ellagic acid (34, 49). Selonsertib significantly decreases the substrate accessibility of CDK6 by acting as a competitive inhibitor which eventually results in enzyme inhibition. Selonsertib may be considered as a drug of interest to target CDK6 for the therapeutic management of associated diseases.

## Conclusion

In conclusion, our study signifies that selonsertib could be a potent inhibitor of CDK6. It shows strong binding affinity, kinase inhibition, and several non-covalent interactions with the substrate-binding pocket are formed. Targeting CDK6 by selonsertib could be a promising therapeutic approach for cancer and other CDK6 associated disease therapy. Overall, our results encourage future researchers to explore using selonsertib in developing potent and selective CDK6 inhibitors for the clinical management of related anomalies. This study can be a stepping stone for further evaluation to explore the possibility of using selonsertib to address CDK6-related human pathologies.

## Data Availability Statement

The original contributions presented in the study are included in the article/supplementary material. Further inquiries can be directed to the corresponding authors.

## Author Contributions

Conceptualization, MHB, MY, JJD and MIH; methodology, MHB, MY, MIK, and MYA, software, MIK, IK and IA; validation, MIK, MH, MY, IK, and JJD; formal analysis, MIK, MY, and MIH; investigation, MIK, IK and IA; resources, MHB and JJD; data curation,MHB, and MY; writing —original draft preparation, MHB and MY; writing—review and editing, MIH and JJD; visualization, MIK and MYA; supervision, MH; project administration, MIH and JJD; funding acquisition, MYA and JJD. All authors have read and agreed to the published version of the manuscript.

## Funding

National Research Foundation of Korea (Grant Number: NRF-2021K1A4A7A02098793).

## Conflict of Interest

The authors declare that the research was conducted in the absence of any commercial or financial relationships that could be construed as a potential conflict of interest.

## Publisher’s Note

All claims expressed in this article are solely those of the authors and do not necessarily represent those of their affiliated organizations, or those of the publisher, the editors and the reviewers. Any product that may be evaluated in this article, or claim that may be made by its manufacturer, is not guaranteed or endorsed by the publisher.
